# Optimization of COVID-19 prevention and control measures during the Beijing 2022 Winter Olympics: a model-based study

**DOI:** 10.1186/s40249-022-01019-2

**Published:** 2022-09-06

**Authors:** Lingcai Kong, Mengwei Duan, Jin Shi, Jie Hong, Xuan Zhou, Xinyi Yang, Zheng Zhao, Jiaqi Huang, Xi Chen, Yun Yin, Ke Li, Yuanhua Liu, Jinggang Liu, Xiaozhe Wang, Po Zhang, Xiyang Xie, Fei Li, Zhaorui Chang, Zhijie Zhang

**Affiliations:** 1 Department of Mathematics and Physics, North China Electric Power University, Baoding, 071003 China; 2 Hebei Key Laboratory of Physics and Energy Technology, North China Electric Power University, Baoding, 071003 China; 3 Department of Epidemiology and Health Statistics, Fudan University, Shanghai, 200032 China; 4 Department of Power Engineering, North China Electric Power University, Baoding, 071003 China; 5 Division of Infectious Disease, Key Laboratory of Surveillance and Early-Warning On Infectious Disease, Chinese Center for Disease Control and Prevention, Beijing, China

**Keywords:** Dynamic model, The Beijing 2022 Winter Olympics, Prevention and control measure, COVID-19

## Abstract

**Background:**

The continuous mutation of severe acute respiratory syndrome coronavirus 2 has made the coronavirus disease 2019 (COVID-19) pandemic complicated to predict and posed a severe challenge to the Beijing 2022 Winter Olympics and Winter Paralympics held in February and March 2022.

**Methods:**

During the preparations for the Beijing 2022 Winter Olympics, we established a dynamic model with pulse detection and isolation effect to evaluate the effect of epidemic prevention and control measures such as entry policies, contact reduction, nucleic acid testing, tracking, isolation, and health monitoring in a closed-loop management environment, by simulating the transmission dynamics in assumed scenarios. We also compared the importance of each parameter in the combination of intervention measures through sensitivity analysis.

**Results:**

At the assumed baseline levels, the peak of the epidemic reached on the 57th day. During the simulation period (100 days), 13,382 people infected COVID-19. The mean and peak values of hospitalized cases were 2650 and 6746, respectively. The simulation and sensitivity analysis showed that: (1) the most important measures to stop COVID-19 transmission during the event were daily nucleic acid testing, reducing contact among people, and daily health monitoring, with cumulative infections at 0.04%, 0.14%, and 14.92% of baseline levels, respectively (2) strictly implementing the entry policy and reducing the number of cases entering the closed-loop system could delay the peak of the epidemic by 9 days and provide time for medical resources to be mobilized; (3) the risk of environmental transmission was low.

**Conclusions:**

Comprehensive measures under certain scenarios such as reducing contact, nucleic acid testing, health monitoring, and timely tracking and isolation could effectively prevent virus transmission. Our research results provided an important reference for formulating prevention and control measures during the Winter Olympics, and no epidemic spread in the closed-loop during the games indirectly proved the rationality of our research results.

**Graphical Abstract:**

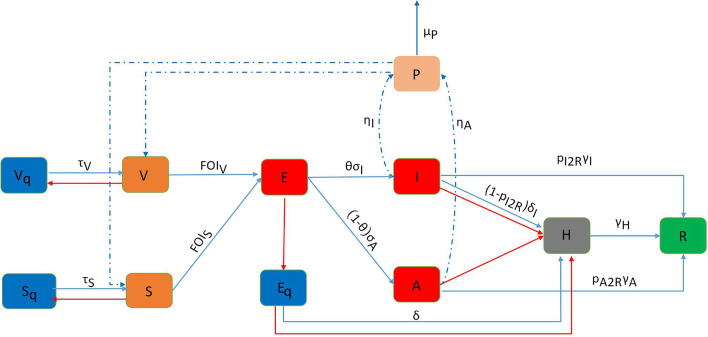

**Supplementary Information:**

The online version contains supplementary material available at 10.1186/s40249-022-01019-2.

## Background

Coronavirus disease 2019 (COVID-19) has been reported in over 200 countries in the last 2 years. As a “global pandemic” [1], it has not only posed a serious threat to human health but also had profound consequences on various aspects, including the economy, public psychology, the sports industry and even air quality [2, 3]. COVID-19 has caused the suspension, cancellation, or postponement of almost all major sports events globally, causing huge economic losses to event organizers, athletes, sponsors, broadcasters, and other stakeholders. To minimize the impact on the sporting industry, with the effects of COVID-19 gradually weakened, many sports events have resumed. From July to September 2021, the 2020 Tokyo Olympic Games and Paralympics, postponed for 1 year, were held, during which 41 athletes and 822 non-athletes (Olympic-related) tested positive [4]. Compared with the new cases reported in Japan during the same period, there were very few cases of infection among Olympic-related personnel, and the overall control strategy was deemed to be relatively successful [4]. However, the prevention and control strategies used were not perfect, and it is worth examining whether these measures can be further optimized for future events.

Due to the COVID-19 epidemic, a significant amount of challenging work has been done on epidemic prevention and safety [5]. First, the spread of the Delta and Omicron strains brought about uncertainty in that the Delta and Omicron variant are more contagious than the original strain [6, 7]. In some other countries, the epidemic was severe. Furthermore, each country has different prevention and control strategies, which caused a number of difficulties for China as the host of the Beijing 2022 Winter Olympics. Second, the Olympic Games had the following characteristics: (i) The personnel came from different countries and included athletes, coaches, journalists, team officials, venue staff, and volunteers. (ii) Many places were involved, such as the Olympic Village, competition venues, and training venues. (iii) Many steps were involved: including the entry and exit of personnel, accommodation, transportation, catering, competitions, and the opening and closing ceremonies [8]. These characteristics made it more difficult to prevent and control the spread of COVID-19. Finally, during the Beijing 2022 Winter Olympics and Winter Paralympics, as many people from different countries and regions entered China and gathered in Beijing and Zhangjiakou Cities, there was a high probability of a certain number of positive cases.

Controlling the spread of COVID-19 was key to the successful hosting of the Winter Olympics. This was achieved with accurate and effective epidemic prevention and control measures. Excessively strict measures would affect the competition experience and progress, while excessively loose measures might make the Winter Olympics a hotbed for the spread of COVID-19. Therefore, balancing “excitement” and “safety” was necessary when formulating the prevention and control measures. This is a challenge that other large-scale sports events during the pandemic will also encounter. The Chinese government adheres to the principle that people are supreme and life is supreme. In August 2021, China began to implement the epidemic prevention policy of “dynamic clearing” in accordance with the overall prevention and control strategy of “preventing import from abroad and rebound in the country” [9]. This practice has effectively guaranteed the sustained and stable development of China’s economy and society and ensured the health and safety of people in the country. For the Beijing Winter Olympics and Winter Paralympics to be successful, we needed to continue to implement the Chinese government’s epidemic prevention principles and requirements, strengthen key measures, identify cases early and reduce infection, and keep safety as the focus. In the preparatory stage of the prevention and control measures for the 2022 Beijing Winter Olympics and Winter Paralympics, we established a COVID-19 dynamic model with pulse detection and isolation effect based on the prevention and control requirements of the Winter Olympics and Winter Paralympics, designed different scenarios to reflect the intensity or frequency of prevention and control measures, analyzed and evaluated the effects of different measures on controlling the spread of COVID-19 by simulating its spread under various scenarios, and analyzed the sensitivity of key parameters in the effective measures. This provided an important scientific basis for optimizing the formulation of epidemic prevention and control measures during the games and ensuring the safe and smooth hosting of the Beijing Winter Olympics and Winter Paralympics. Now, the 24th Beijing Winter Olympics and Winter Paralympics have achieved complete success. During the Games, there was no epidemic spread in the closed loop [10]. The prevention of COVID-19 at the games was highly praised by the International Olympic Committee as well as many media outlets in China and other countries [11]. This indirectly shows the effectiveness of the prevention and control measures implemented. This paper introduces our quantitative evaluation and optimization of the prevention and control measures based on the model at the preparatory stage of the games to provide ideas and methods that could be used at other sports events in the future.

## Methods

### Introduction of the model

Based on the characteristics of the short time period, closed-loop management, and sufficient medical resources of the Winter Olympics, this study established a deterministic dynamic model with pulse detection and isolation effect based on the following:

(1) Did not consider natural birth and death.

(2) Did not consider death due to illness.

(3) Infected people will not be re-infected during the event after recovery.

(4) After positive cases are detected, their close contacts are quickly tracked and isolated, ignoring time delays.

(5) Considered environmental transmission.

**Fig. 1:**
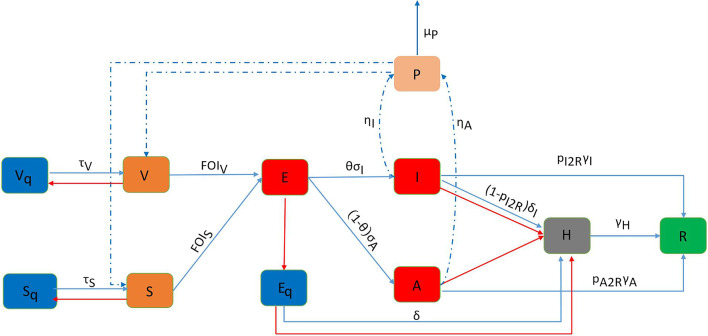
Compartmental model structure with pulse detection and isolation effect. The blue arrows indicate routine transfer. The red arrows indicate impulse transfer. The dotted line indicates that the patient discharges the virus into the environment and that susceptible people become infected by the virus in the environment. $FO{I}_{V}=(c{p}_{SC}\frac{I+A}{N}+{\beta }_{SE}\frac{P}{N}){(1-f}_{S})p, FO{I}_{S}=c{p}_{SC}\frac{I+A}{N}+{\beta }_{SE}\frac{P}{N}$. We consider susceptible ($S$), exposed ($E$), symptomatic infected ($I$), asymptomatic infected ($A$), hospitalized ($H$), recovery ($R$), isolated (${V}_{q}$, ${S}_{q}$, ${E}_{q}$) individuals and the virus in the environment ($P$), more details of the model and the transitions between compartments are provided in the text and Table 1

Combining the transmission characteristics of severe acute respiratory syndrome coronavirus 2 (SARS-CoV-2), we established the COVID-19 transmission dynamics model based on the classic SEIR model [12]. The structure of the dynamic model compartment is shown in Fig. 1, with further details in Table 1. On testing days, the model can be expressed by the following equations in impulse form (the differential equations of non-testing days can be seen in Additional file 1):

**Table 1:** Description of the model compartments

Compartment	Description
$S$	Susceptible individuals
$E$	Exposed individuals (infected but not infectious)
$A$	Asymptomatic infected persons with infectious ability
$I$	Symptomatic infected persons with infectious ability
$R$	Recovered individuals, get antibodies after recovery
$V$	Vaccinated individuals, finished the whole course of COVID-19 vaccination but some did not produce antibodies, still susceptible
${S}_{q}$	Quarantined susceptible individuals, close contacts who are not actually infected and will not be infected during the quarantine period
${V}_{q}$	Quarantined vaccinated individuals, close contacts who are vaccinated and are not actually infected, will not be infected during quarantine
${E}_{q}$	Quarantined exposed individuals, close contacts who are infected but not yet infectious, will be identified through close contact tracing of the infected person and admitted to hospital after testing positive
$H$	Hospitalized individuals, infected person (whether asymptomatic or not) admitted to hospital after being detected
$P$	Viruses in the environment, excreted by undetected infected individuals, can infect susceptible individuals and vaccinated individuals who have not produced antibodies

When $t={t}_{n}({t}_{n}={t}_{0}+nT$) (${t}_{0}$ is the initial time, $T$ is the interval between two nucleic acid tests, ${t}_{n}$ is the test time),

1 $$\begin{array}{c}S\left({t}^{+}\right)=S\left({t}^{-}\right)-n*{p}_{S}*{p}_{C}*q*\left(A\left({t}^{-}\right)\left(1 - {p}_{A2R}\right)+I\left({t}^{-}\right)\left(1 - {p}_{I2R}\right)\right) \end{array}$$

2 $$\begin{array}{c}{S}_{q}\left({t}^{+}\right)={S}_{q}\left({t}^{-}\right)+n*{p}_{S}*{p}_{C}*q*\left(A\left({t}^{-}\right)\left(1 - {p}_{A2R}\right)+I\left({t}^{-}\right)\left(1 - {p}_{I2R}\right)\right)\end{array}$$

3 $$\begin{array}{c}V\left({t}^{+}\right)=V\left({t}^{-}\right)-n*{p}_{V}*{p}_{C}*q*\left(A\left({t}^{-}\right)\left(1 - {p}_{A2R}\right)+I\left({t}^{-}\right)\left(1 - {p}_{I2R}\right)\right)\end{array}$$

4 $$\begin{array}{c}{V}_{q}\left({t}^{+}\right)={V}_{q}\left({t}^{-}\right)+n*{p}_{V}*{p}_{C}*q*\left(A\left({t}^{-}\right)\left(1 - {p}_{A2R}\right)+I\left({t}^{-}\right)\left(1 - {p}_{I2R}\right)\right)\end{array}$$

5 $$\begin{array}{c}E\left({t}^{+}\right)=E\left({t}^{-}\right)-n*{p}_{E}*{p}_{C}*q*\left(A\left({t}^{-}\right)\left(1 - {p}_{A2R}\right)+I\left({t}^{-}\right)\left(1 - {p}_{I2R}\right)\right)-{q}_{E}E\left({t}^{-}\right)\end{array}$$

6 $$\begin{array}{c}{E}_{q}\left({t}^{+}\right)={E}_{q}\left({t}^{-}\right)+n*{p}_{E}*{p}_{C}*q*\left(A\left({t}^{-}\right)\left(1 - {p}_{A2R}\right)+I\left({t}^{-}\right)\left(1 - {p}_{I2R}\right)\right)+{q}_{E}E\left({t}^{-}\right)\end{array}$$

7 $$\begin{array}{c}I\left({t}^{+}\right)=I\left({t}^{-}\right)-q*I\left({t}^{-}\right)\left(1 - {p}_{I2R}\right)\end{array}$$

8 $$\begin{array}{c}A\left({t}^{+}\right)=A\left({t}^{-}\right)-q*A\left({t}^{-}\right)\left(1 - {p}_{A2R}\right)\end{array}$$

9 $$\begin{array}{c}H\left({t}^{+}\right)=H\left({t}^{-}\right)+q*\left(I\left({t}^{-}\right)\left(1 - {p}_{I2R}\right)+A\left({t}^{-}\right)\left(1 - {p}_{A2R}\right)\right)\end{array}$$

10 $$\begin{array}{c}R\left({t}^{+}\right)=R\left({t}^{-}\right)\end{array}$$

11 $$\begin{array}{c}P\left({t}^{+}\right)=P\left({t}^{-}\right)\end{array}$$

Among them, ${t}^{-}$ represents the time before nucleic acid testing, and ${t}^{+}$ represents the time after testing, tracking and isolation of close contacts.

$S$ represents the susceptible individuals who have not been vaccinated, and $V$ represents those who have finished the whole course of COVID-19 vaccination. Because the COVID-19 vaccine protection rate has not reached 100%, some vaccinated people are still susceptible to SARS-CoV-2. Susceptible individuals and those who have been vaccinated but have not produced antibodies become exposed individuals ($E$, without infectivity) after being infected by infected individuals (with or without symptoms) and then develop into infected individuals with infectivity after the incubation period. These people can be divided into symptomatic infected individuals ($I$) and asymptomatic infected individuals ($A$). Infected individuals can be detected through regular nucleic acid testing, and they will be sent to the hospital immediately after being detected and be classified as hospitalized ($H$); Symptomatic infected individuals may also enter $H$ after being detected through health monitoring. After treatment and recovery, the patients are classified as recovered ($R$). Infected individuals (especially asymptomatic infected people) may not be detected, and after the virus disappears from their bodies, they will naturally recover and be classified as recovered ($R$). Once a case is detected, the epidemic prevention and control staff will immediately track their close contacts and implement isolation measures. ${V}_{q}$, ${S}_{q}$, and ${E}_{q}$ respectively refer to the isolation of those who have completed vaccination, who are susceptible and exposed, and the close contacts that are isolated according to their actual states. Note that, some exposed individuals ($E$) found by nucleic acid testing are also included in ${E}_{q}$. Isolated exposed individuals (${E}_{q}$) will be sent to the hospital and be classified as hospitalized ($H$) after being diagnosed by nucleic acid testing. The model also considers that the virus spreads to people through the environment [13]. Undetected infected individuals ($A$, $I$) pollute the surrounding environment and surfaces by discharging droplets containing the virus, and susceptible individuals are infected by coming into contact with the contaminated environment or surfaces.

The key principles of the *Epidemic Prevention Manual* include vaccination, closed-loop management, nucleic acid testing, tracking and isolation of close contacts, isolation treatment of cases, health monitoring, contact reduction, and hygiene awareness. Among them, the formulation of key prevention and control measures was based on the dynamic model established in this study. Compared with the existing COVID-19 dynamic models, the model established in this study has these characteristics:

1. It is assumed that the newly discovered cases and their close contacts will be quickly isolated, ignoring the time delay and using a dynamic model with impulse effect.
2. In addition to being found through regular nucleic acid testing, symptomatic infected individuals ($I$) can also be found through health monitoring.
3. Environmental infection is considered: undetected infected individuals ($A$, $I$) discharge droplets with the virus through sneezing, breathing, polluting the environment or articles that have been in close contact with them, resulting in infection of susceptible persons in the same environment or in contact with contaminated articles.
4. It is assumed that the medical resources are adequate, and all cases are sent to the hospital for treatment.

The description of the parameters in the model and equation are shown in Table 2.

**Table 2:** Description of model parameters and their values

Parameters	Description	Value (range)	Source
$c$	Number of contacts of an individual per unit time	10 (5–20)	[28]
${p}_{sc}$	The transmission rate of the virus when a susceptible person contacts an infected person	0.5 (0.4–0.7)	[29]
$p$	The ratio of susceptibility of vaccinated individuals to symptomatic individuals	0.8	Assumed
${\beta }_{SE}$	Effective contact rates of susceptible individuals with viruses in the environment	0.00414 (0.001–0.01)	[30]
${f}_{s}$	The effective protection rate of the vaccine	68.7% (58.1–76.7%)	[31]
${\tau }_{S}$	Rate of quarantined susceptible individual releasing from quarantine, reciprocal of its average quarantine time	1/7	Assumed
${\tau }_{V}$	Rate of quarantined vaccinated individual releasing from quarantine, reciprocal of its average quarantine time	1/7	Assumed
$\theta$	Proportion of infected people with symptoms	0.8 (0.5–0.9)	China CDC
${\sigma }_{I}$	Reciprocal of the average latent period of symptomatic infections	1/4.0 (1/4.4–1/3.5)	[32]
${\sigma }_{A}$	Reciprocal of the average latent period of asymptomatic infections	1/4.0 (1/4.4–1/3.5)	[32]
${\delta }_{I}$	The transfer rate of symptomatic infected individuals who were detected and admitted to hospital through health monitoring, reciprocal of the average time from being infectious to detection through health monitoring	1/2	Assumed
$\delta$	The transfer rate of quarantined exposed individuals who were detected and admitted to hospital through health monitoring	1/4	Assumed
${\gamma }_{I}$	The natural recovery rate of symptomatic infected individuals, the reciprocal of infectious period in symptomatic infected	1/10 (1/14–1/7)	China CDC
${\gamma }_{A}$	The natural recovery rate of asymptomatic infected individuals, the reciprocal of infectious period in asymptomatic infected	1/7 (1/10–1/5)	China CDC
${p}_{I2R}$	Proportion of symptomatic infected persons who were not detected and recovered spontaneously	0.025	Assumed
${p}_{A2R}$	Proportion of asymptomatic infected persons who were not detected and recovered spontaneously	0.025	Assumed
${\gamma }_{H}$	The average recovery rate of hospitalized individuals, reciprocal of hospitalization period	1/14 (1/20–1/10)	China CDC
${\eta }_{A}$	The rate at which asymptomatic infected individuals shed virus into the environment	0.05 (0.01–0.1)	[30]
${\eta }_{I}$	The rate at which symptomatic infected individuals shed virus into the environment	0.1 (0.02–0.2)	[30]
${\mu }_{P}$	Virus mortality in the environment, the reciprocal of the average virus survival time	1/5 (1/10–1)	[30]
$q$	Proportion of infected individuals detected by nucleic acid testing	0.8	[33]
${q}_{E}$	Proportion of exposed individuals detected by nucleic acid testing	0.4	Assumed
$n$	Number of close contacts per infected individual	50	Assumed
$k$	Frequency of nucleic acid testing	3	Assumed
${p}_{C}$	Probability that close contacts are successfully traced and isolated	0.9	[34]
${p}_{V}$	Proportion of close contacts who are vaccinated individuals	Determined by the ratio of $V/N$ before nucleic acid testing	
${p}_{S}$	Proportion of close contacts who are susceptible individuals	Determined by the ratio of $S/N$ before nucleic acid testing	
${p}_{E}$	Proportion of close contacts who are exposed individuals	Determined by the ratio of $E/N$ before nucleic acid testing	

### Scenario setting

To quantify the effects of different prevention and control measures, we set up different scenarios and simulated the spread of COVID-19 in each scenario. Firstly, we set the baseline level of each prevention and control measure at a moderate intensity and then strengthened or relaxed different prevention and control measures to set up comparable control scenarios:

(1) Scenario 1 (baseline level, S1): It is assumed that the initial total number of people is 50,000, among which 80% are vaccinated and 20% are unvaccinated susceptible people. It is assumed that five exposed people ($E$) are introduced at the initial time point, and the frequency of nucleic acid testing is once every three days. Refer to Table 2 for the values of the other parameters.

(2) Scenarios 2 and 3 (S2, S3): These reflect the strictness of the entry (closed-loop entry) policy, including vaccination before entry, remote prevention and control, nucleic acid testing before and after entry, and isolation of unvaccinated people, which mainly reflects the number of infected or exposed people at entry.

(3) Scenarios 4 and 5 (S4, S5): These reflect the compliance to the principle of “contact reduction,” including closed-loop management, maintaining social distance, avoiding closed environments and crowded people, wearing masks, avoiding hugs and contact, and strict hygiene awareness, which is mainly reflected in the effective contact rate and the number of close contacts per infected person. The effective contact rate is $\beta =c*{p}_{sc}$ and the values adopted in this study are set as S4 and S5.

(4) Scenarios 6 and 7 (S6, S7): These reflect the risk of environmental transmission, such as reducing virus discharge by wearing a mask and reducing virus load in the environment by regular disinfection and ventilation, which are mainly reflected in virus load and survival time in the environment.

(5) Scenarios 8 and 9 (S8 and S9): These reflect the effectiveness of nucleic acid testing frequency and health monitoring measures, mainly reflecting the timeliness of case detection.

See Additional file 1 for the parameter values in each scenario.

### Evaluation indicators

We compared the number of daily new cases, the cumulative number of cases, the current number of patients, the average number of inpatients, and the peak number of inpatients of COVID-19 in different scenarios and analyzed the spread of COVID-19 in different scenarios.

## Results

### Baseline levels

**Fig. 2:**
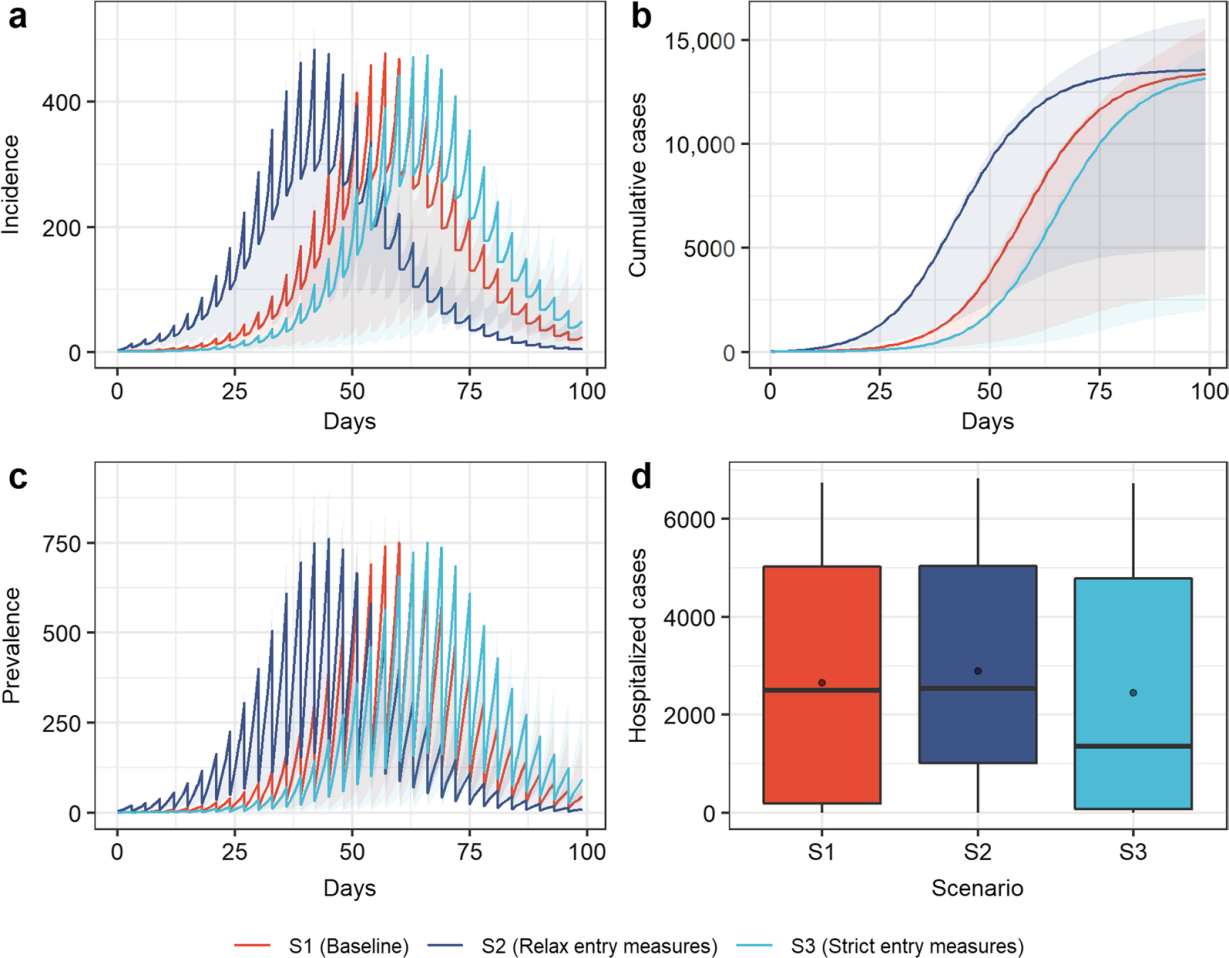
Simulation results of COVID-19 transmission in scenarios 2 (relax entry measures), scenarios 3 (strict entry measures), and baseline. **a** Incidence over time; **b** cumulative cases over time; **c** prevalence over time; **d** hospitalized cases. Boxplots represent the mean (dots), median (black line), quarter and third quartile (upper and lower edges of the box), maximum (upper edge) and minimum (lower edge) of hospitalized cases for each scenario

**Fig. 3:**
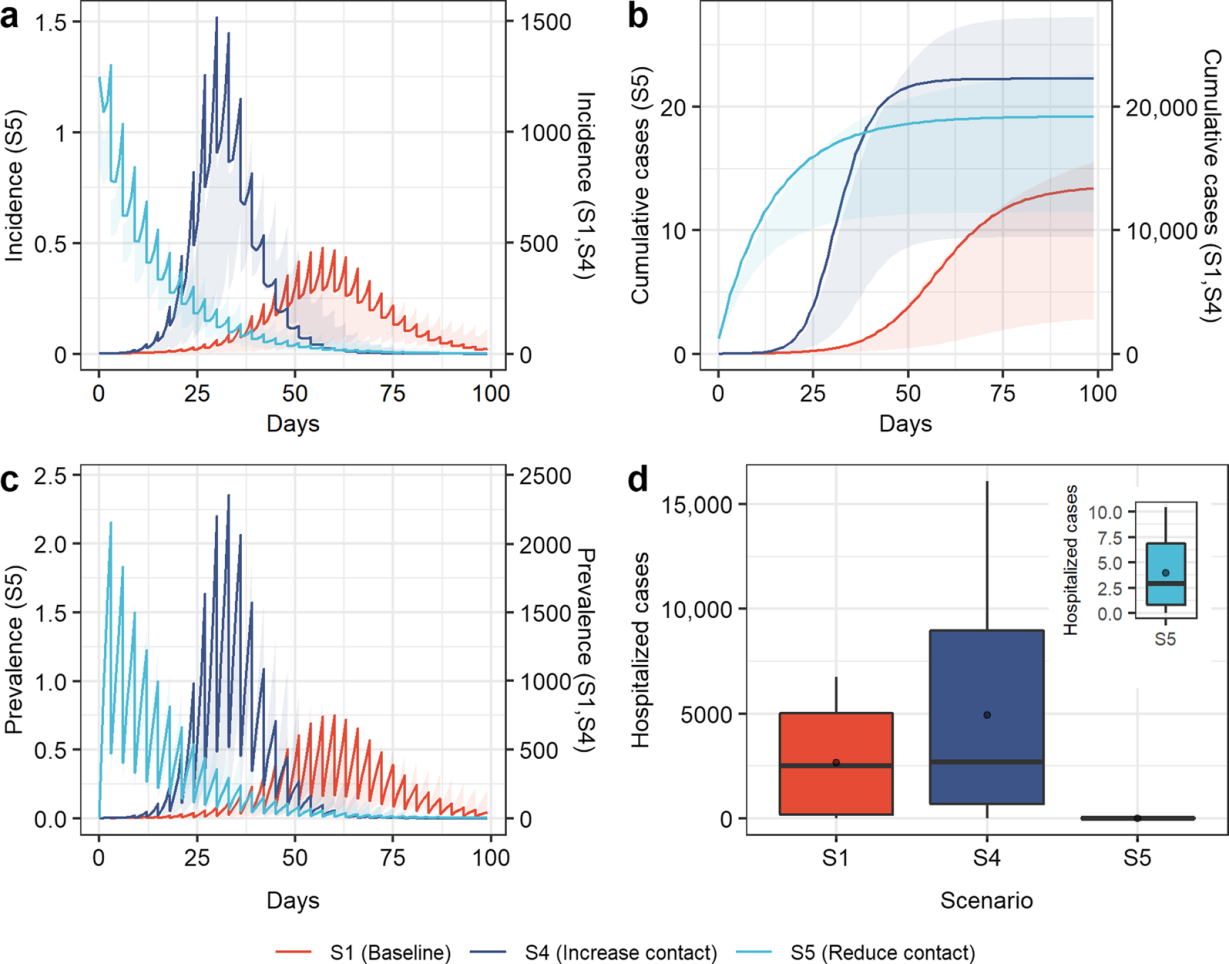
Simulation results of COVID-19 transmission in scenarios 4 (increase contact), scenarios 5 (reduce contact), and baseline. **a** Incidence over time; **b** cumulative cases over time; **c** prevalence over time; **d** hospitalized cases. Boxplots represent the mean (dots), median (black line), quarter and third quartile (upper and lower edges of the box), maximum (upper edge) and minimum (lower edge) of hospitalized cases for each scenario

**Fig. 4:**
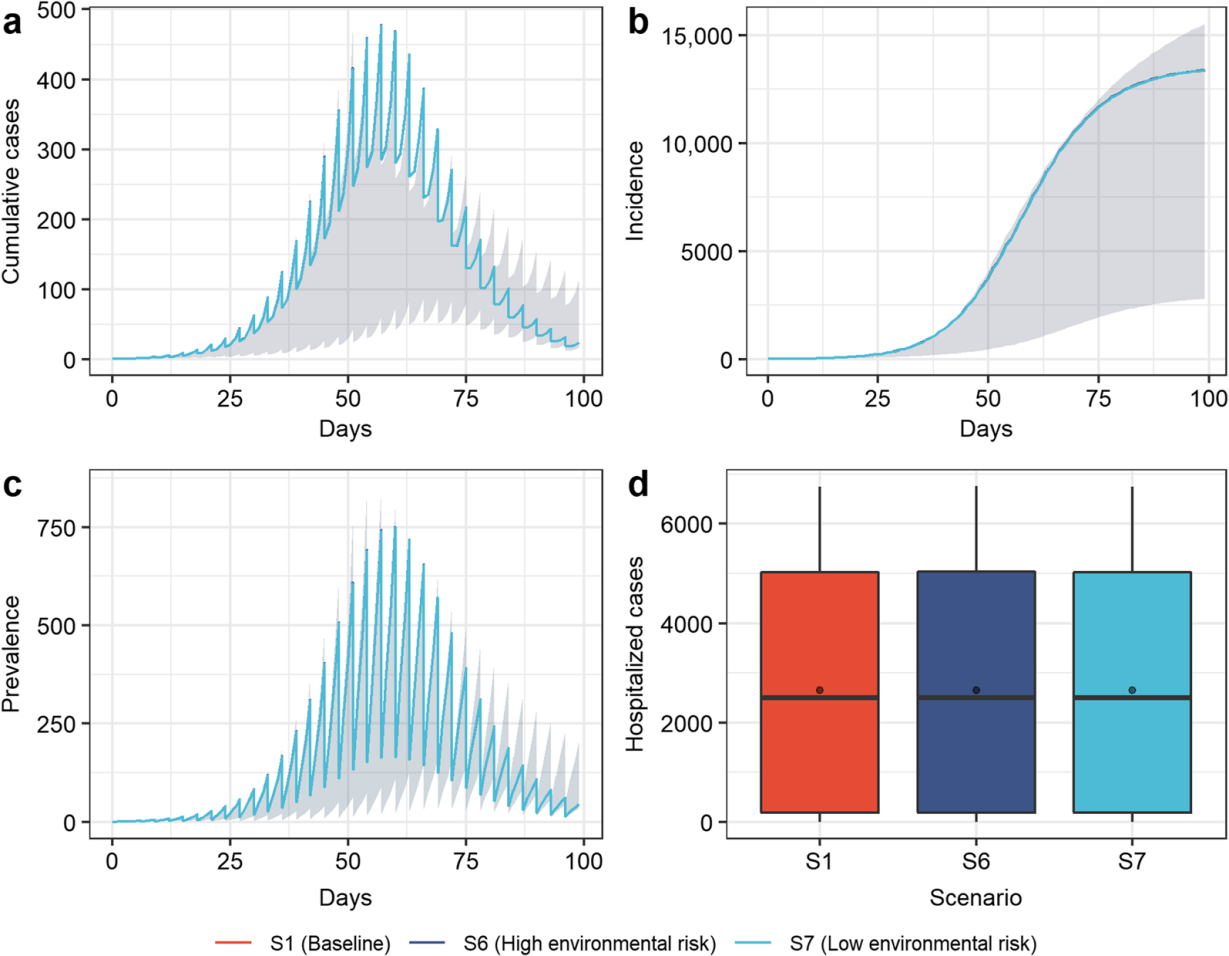
Simulation results of COVID-19 transmission in scenarios 6 (high environmental risk), scenarios 7 (low environmental risk), and baseline. **a** Incidence over time; **b** cumulative cases over time; **c** prevalence over time; **d** hospitalized cases. Boxplots represent the mean (dots), median (black line), quarter and third quartile (upper and lower edges of the box), maximum (upper edge) and minimum (lower edge) of hospitalized cases for each scenario. In figure **a**–**c** the simulated curves in the above scenarios basically overlap

**Fig. 5:**
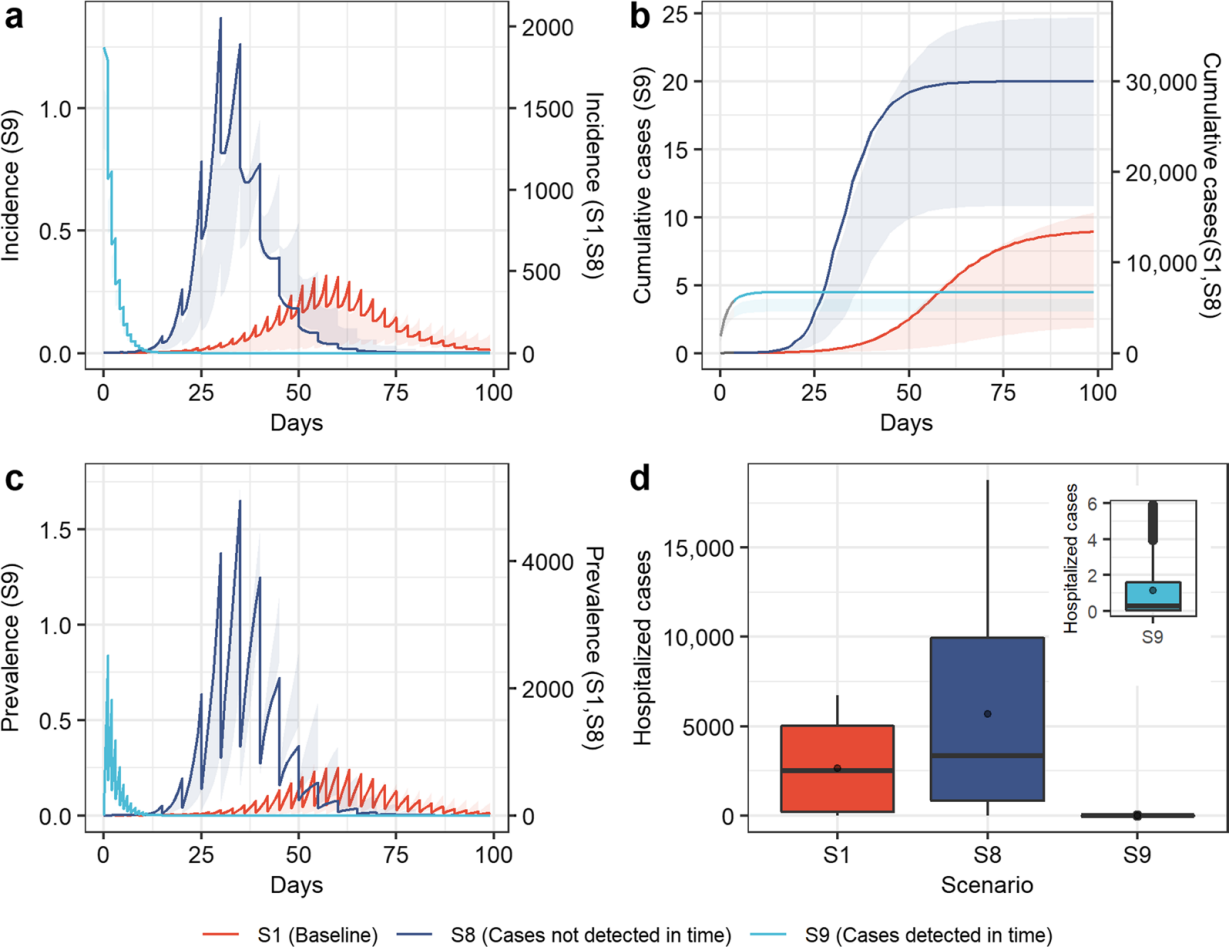
Simulation results of COVID-19 transmission in scenarios 8 (cases not detected in time), scenarios 9 (cases detected in time), and baseline. **a** Incidence over time; **b** cumulative cases over time; **c** prevalence over time; **d** hospitalized cases. Boxplots represent the mean (dots), median (black line), quarter and third quartile (upper and lower edges of the box), maximum (upper edge) and minimum (lower edge) of hospitalized cases for each scenario

Figures 2, 3, 4 and 5 describe the progress of the epidemic at the assumed baseline levels, with an initial population of 50,000, among which 80% had been vaccinated and 20% unvaccinated. Initially, five exposed people ($E$) were introduced into the closed-loop, the nucleic acid testing was carried out once every three days, and the other measures were implemented at moderate intensity (see Additional file 1 for parameters at baseline levels). Due to the effect of pulse detection and isolation, new and existing cases showed a pulse-like decline after being detected and isolated with the nucleic acid testing cycle. The daily number of new cases and the number of current cases peaked on the 57th day, with the peak values of 477 and 750, respectively (Fig. 2a and c). During the simulation period (100 days), 13,382 people contracted COVID-19 (Fig. 2b). The mean and peak values of hospitalized cases were 2650 and 6746, respectively (Fig. 2d).

### Entry measures

The simulation results showed the impact of the strictness of entry measures (Fig. 2). The results showed that strict entry measures such as remote prevention and control, vaccination before entry, and nucleic acid testing could not significantly reduce infection (Fig. 2a–c). However, compared with the baseline level (introduction of five exposed people), the more relaxed entry measures (scenario 2) would cause the peak of the epidemic to appear 15 days earlier, while the implementation of strict entry measures (scenario 3) would delay the peak time of the epidemic by nine days and reduce the average number of hospitalized cases by 7.86% (Fig. 2d).

### Reducing contact

Measures related to “reducing contact” could effectively reduce the scale of virus transmission (Fig. 3). Increasing contact (scenario 4) led to the daily peak of new cases increasing to 3.17 times the baseline level, the epidemic peak occurring 27 days earlier (Fig. 3a and c), the cumulative number of patients increasing by 66.4% (Fig. 3b) and the peak number of hospitalized cases reaching 16,070, 2.38 times that at baseline (Fig. 3d). In contrast, when relevant measures to reduce contact were strictly implemented (scenario 5), the epidemic would be controlled before it caused large-scale spread, and the number of daily new cases and current cases would start to decrease after the first round of nucleic acid testing (the third day), with a cumulative incidence of 19 cases and a peak number of hospitalized cases of only ten cases (0.15% of the baseline level).

### Risk of environmental transmission

It can be seen in Fig. 4 that the risk of environmental transmission of SARS-CoV-2 was very low, and the simulation results of the incidence and number of hospitalized cases in scenarios 6 and 7 coincide with the baseline level. Reducing the risk of environmental transmission based on the baseline level (scenario 7) only reduced the cumulative incidence by 6 cases (0.05%).

### Nucleic acid testing and health monitoring

The influence of the implementation frequency or intensity of nucleic acid testing, health monitoring, and other measures on the spread of COVID-19 was shown in Fig. 5. The simulation results showed that the peak values of daily new cases and current cases in scenario 8 were 4.3 times and 6.6 times the baseline levels, respectively, and the epidemic peak occurred 27 days earlier than the baseline level (Fig. 5a and c). The peak of hospitalized cases in the simulation period was 18,772, which was 2.78 times the baseline level (Fig. 5d). If cases were detected in time through more frequent nucleic acid testing and health monitoring (scenario 9), the daily number of new cases would start to decrease from the first day, with only seven cumulative cases (Fig. 5b) and only six peak hospitalized cases, effectively reducing the infection risk and medical care burden.

### Parameter sensitivity analysis

In this section, we analyze the sensitivity of the parameters from the effective measures (scenarios 5 and 9) to analyze the importance of the corresponding parameters in the combination of measures.

**Fig. 6:**
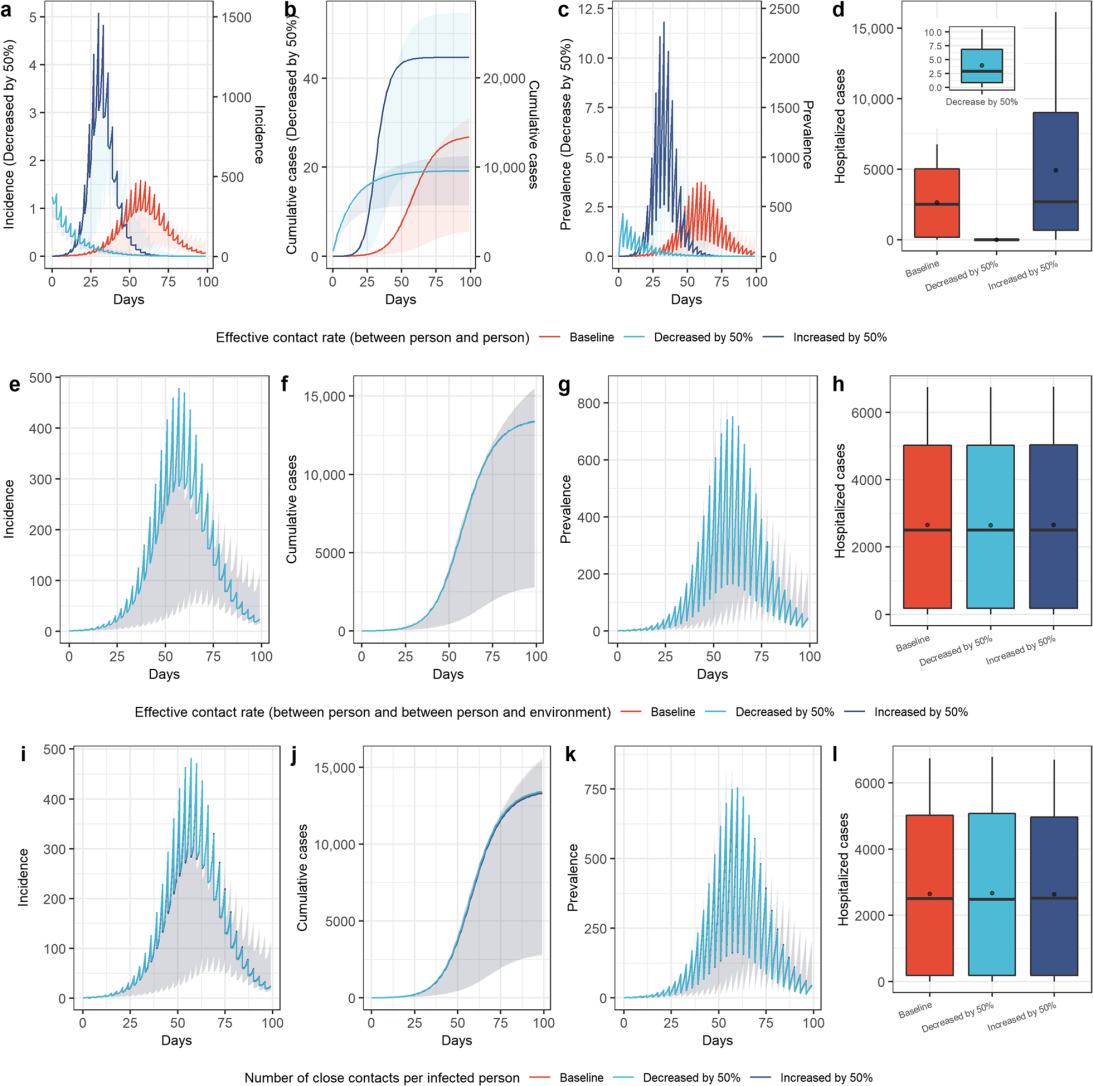
Sensitivity analysis of parameters related to contact. **a**–**d** Sensitivity analysis of effective contact rate between people; **e**–**h** sensitivity analysis of effective contact rate between people and environment; **i**–**l** sensitivity analysis of the number of close contacts per infected person. We simulated the incidence, cumulative cases, and prevalence over time and the hospitalized cases when the three parameters increased by 50% and decreased by 50%, and compared them with the baseline levels. The sensitivity analysis curves of the corresponding parameters in figure **e**, **f**, **g**, **i**, **j**, **k** basically overlap

The sensitivity analysis results of the parameters related to “reducing contact” (effective contact rate between people, and between people and the environment, as well as the number of close contacts per infected person) are shown in Fig. 6. It can be seen that when the effective contact rate between people ($c{p}_{SC}$, $c{pp}_{SC}$) increased by 50%, the peak daily new cases increased to 3.19 times the baseline level, and the epidemic peak occurred 27 days earlier, with the cumulative number of cases reaching 22,308, an increase of 67% over the baseline level (Fig. 6a). The number of hospitalized cases also increased significantly, and its peak value increased to 2.39 times the baseline level (Fig. 6d). When the effective contact rate between people ($c{p}_{SC}$, $c{pp}_{SC}$) decreased by 50%, it immediately decreased after the first round of nucleic acid testing (the third day), and the cumulative number of patients and peak value of hospitalized cases decreased by more than 99% (Fig. 6d). Figure 6e–l shows that the effective contact rate between people and the environment, as well as the number of close contacts per infected person, had no significant influence on the epidemic spread, and the simulation curves basically coincide with the baseline levels.

**Fig. 7:**
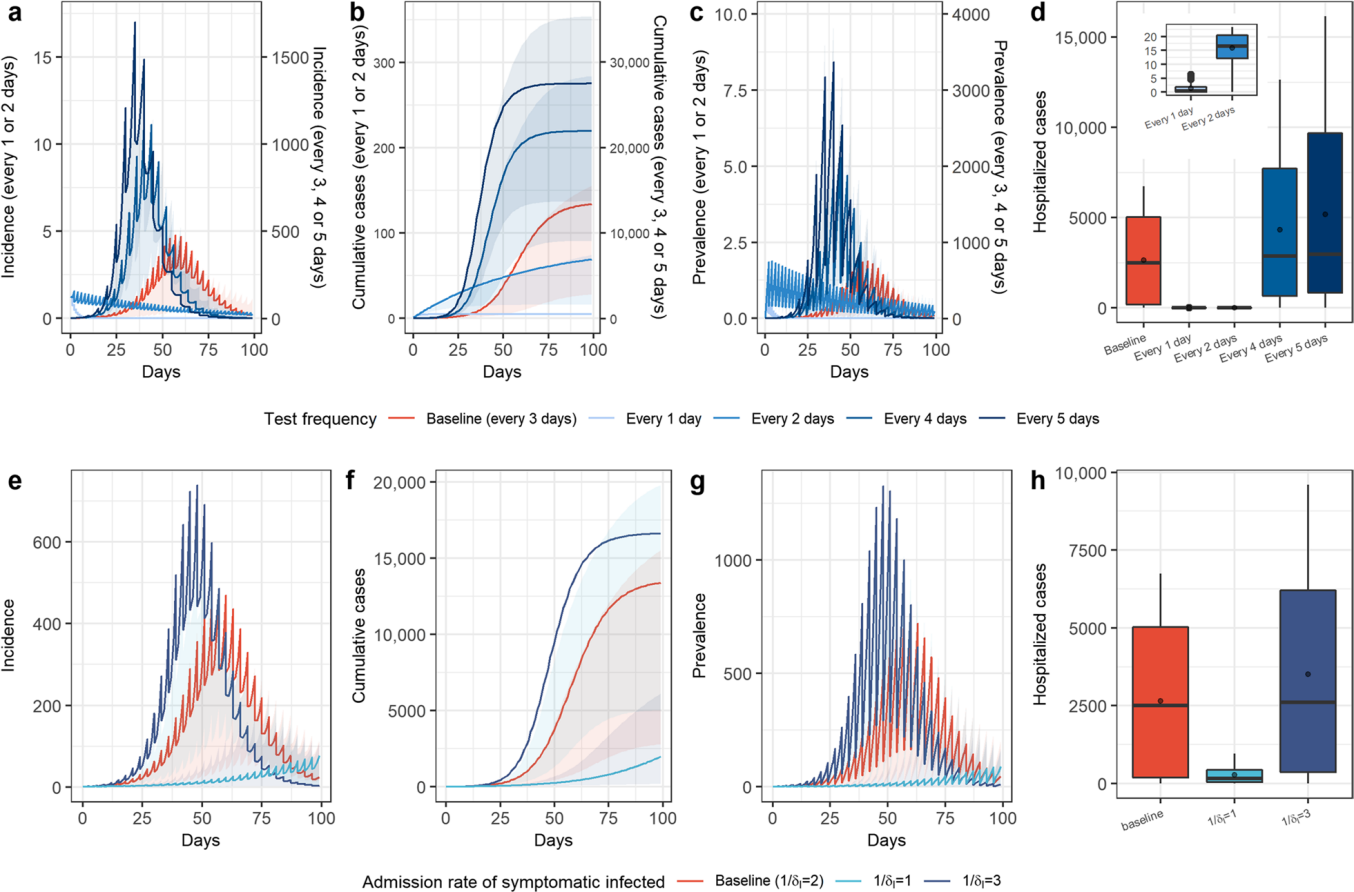
Sensitivity analysis of parameters related to detection of cases. **a**–**d** Sensitivity analysis of nucleic acid. testing frequency, we simulated the incidence, cumulative cases, and prevalence over time and the hospitalized cases when the testing frequency was once a day, every two days, every four days, and every five days, and compared them with the baseline level; **e**–**h** sensitivity analysis of hospital admission rate through health monitoring for symptomatic infection, we simulated the incidence, cumulative cases, and prevalence over time and the hospitalized cases when the time interval ($1/{\delta }_{I}$) from the time when a patient was infectious to the time when the patient was found to be infected through health monitoring was 1 day and 3 days, and compared with the baseline level

The spread of the virus when the nucleic acid testing interval was one to five days was shown in Fig. 7a–d. When the detection frequency was once every one or two days, the epidemic was effectively controlled before large-scale spread, and the cumulative numbers of cases were 5 and 69, respectively (0.04% and 0.52% of the baseline level). If the frequency of nucleic acid testing was reduced to once every four days, the peak of infection occurred 17 days earlier, and the cumulative number of patients and the peak of inpatients increased by 64% and 87%, respectively. When the frequency of nucleic acid testing was reduced to once every five days, the infection speed was faster, the scale was further increased, the peak of infection occurred 22 days earlier, and the cumulative number of patients reached 27,514; an increase of 105% over the baseline level (Fig. 7a–d). Health monitoring could also detect symptomatic infected cases. We used the time interval ($1/{\delta }_{I}$) from the time when a patient was infectious to the time when the patient was found to be infected through health monitoring and admitted to the hospital for treatment to reflect the significance of health monitoring. The simulation results showed that: when the time interval was one day, the infection curve increased very slowly during the simulation period, and the number of inpatients decreased significantly. When the time interval was three days, compared with the baseline level ($1/{\delta }_{I} =2$), the peak values of daily new cases and current cases increased by 55% and 77%, the epidemic peak occurred nine days earlier (Fig. 7e, g), and the cumulative number of patients and peak of inpatients increased by 24% and 42%, respectively (Fig. 7f, h).

## Discussion

The Beijing 2022 Winter Olympics and Winter Paralympics were world-famous large-scale sports events and were seen as an opportunity to show China’s strength and spirit to the world and to present China during the pandemic [14]. However, due to the COVID-19 pandemic, hosting the Winter Olympics and Winter Paralympics was both an opportunity and a challenge. To effectively prevent and control the epidemic while ensuring the smooth hosting of the Winter Olympics was a concern shared by many people. In this paper, the research conducted during the preparatory stage of the events to assist in formulating the prevention and control measures for the Winter Olympics and Winter Paralympics was introduced in detail. The research quantitatively analyzed and evaluated the effectiveness of different measures through scenario simulation, providing an important reference for the formulation of the *Epidemic Prevention Manual for the Beijing 2022 Winter Olympics and Winter Paralympics* (on December 14, 2021, Beijing Organizing Committee for the Winter Olympics issued the second edition, from now on referred to as the Epidemic Prevention Manual) [8]. It was found that the most effective measures to prevent outbreaks of COVID-19 during the Winter Olympics and Winter Paralympics were to detect cases in time through high-frequency nucleic acid testing and daily health monitoring and to track and isolate their close contacts and reduce contact. Second, strict entry measures were found to delay the epidemic peak and provide time for prevention, control, and mobilization of medical resources. During the Olympic Games, SARS-CoV-2 was mainly transmitted from person to person, and the risk of environmental transmission was low.

The simulation results showed that reducing contact could block the transmission chain and thus quickly and effectively control the spread of SARS-CoV-2. Current evidence shows that SARS-CoV-2 mainly spreads among people in close contact with each other and when droplets or aerosols containing the virus are inhaled or come in direct contact with people’s eyes, nose, or mouth [15]. Therefore, reducing contact can reduce the risk of transmission. At the early stage of the COVID-19 pandemic, many countries adopted different degrees of non-pharmaceutical intervention measures such as isolation, reducing or forbidding gatherings, closing schools, encouraging people to work from home, restricting travel, and increasing social distance. These measures directly reduced the contact rate between people. Research has shown that these measures effectively slowed down the spread of the pandemic [16, 17]. In addition, tracking close contacts is also a common measure to prevent COVID-19 from spreading, and reducing contact can improve the probability of successfully tracking close contacts, thus isolating close contacts and stopping the chain of transmission [18, 19]. According to the results of the current research, the basic principles of “closed-loop management” and “contact reduction” are put forward in the *Epidemic Prevention Manual*: all Olympic-related personnel, including relevant staff in China, were required to implement closed-loop management to ensure that they had no contact with the public or people outside the closed-loop; specific measures such as minimizing contact, wearing masks, avoiding closed environments and gatherings as much as possible, and using the special transportation system installed for the Winter Olympics were all aimed at reducing contact. During the 2020 Tokyo Summer Olympics and Paralympics, although the number of COVID-19 cases in Tokyo hit record highs, the infection rate among Olympic-related personnel was very low, and the genome sequencing data of the Japanese government confirmed that COVID-19 did not spread between Olympic-related personnel and local residents [20]. This showed that the series of prevention and control measures taken by the organizers of the Tokyo Olympic Games were relatively successful. One of their core strategies was the bubble scheme, i.e., a series of measures to isolate Olympic-related personnel from the local public and measures to reduce contact, such as the mandatory wearing of masks, and prohibition of spectators, allowed most athletes and staff members to avoid infection [4, 20]. However, the bubble policy was not strictly implemented, and the closed-loop management was not strict when athletes arrived at the airport. The activities of Olympic-related personnel in the closed-loop were unrestricted, and a large number of workers went home to live and did not commute in Olympic-specific vehicles, resulting in two-thirds of the more than 800 Olympic-related workers infected with SARS-CoV-2 being local Japanese workers [21]. This suggested that a closed-loop management system had to be strictly implemented during the Beijing Winter Olympics.

The second key measure highlighted in this study is high-frequency nucleic acid testing and daily health monitoring. High-frequency nucleic acid testing aims to detect cases as early as possible, especially asymptomatic infected people and those still in the incubation period. Frequent health monitoring is conducive to detecting symptomatic infected people early. Early detection of cases directly reduces the risk of transmission and allows the tracking and isolation of close contacts in time, which help break the chain of transmission and prevent the virus from spreading further. High-frequency tests and health monitoring alone cannot stop the spread of COVID-19, and they need to be combined with tracking and isolation measures [19, 22]. According to the results of this study, the basic principle of “detection, tracking, and isolation” was included in the *Epidemic Prevention Manual*, requiring foreign athletes to start monitoring their health status 14 days before coming to China and undergo nucleic acid testing before and at the time of entry. Furthermore, during the competition, nucleic acid testing was required every day. Further testing was required if people had any symptoms or were deemed to be a close contact. High-frequency detection was also considered one of the key measures for the success of epidemic prevention at the Tokyo Olympic Games [4].

This study also found that strict entry measures, or more generally, reducing the number of infected or exposed people entering the closed-loop system, could delay the epidemic peak. It is worth noting that this results from general prevention and control measures. This study aims to compare the influence of different measures on the spread of COVID-19, so it is assumed that a few exposed people enter the closed-loop system. Theoretically, if no infected or exposed people are introduced into a strict closed-loop system, no transmission will occur. Therefore, combining strict entry and other prevention and control measures can timely and effectively prevent and control the spread of the virus. During the Tokyo 2020 Olympics and Paralympics, a total of 41 athletes (all from other countries) were diagnosed with COVID-19, among which 40 (97.6%) were found during the 14-day quarantine period after their arrival [4]. These cases did not cause more athletes to be infected, which shows the importance of strict entry measures.

Finally, we discuss the risk of SARS-CoV-2 spreading through the environment or via surfaces. The simulation showed that the risk of the SARS-CoV-2 virus spreading to people through the environment was low. Considering that SARS-CoV-2 can be transmitted through aerosols [23, 24], many COVID-19 cluster outbreaks have also been related to cold environments [25, 26]. The low-temperature environment at the Winter Olympics would make SARS-CoV-2 survive longer. Therefore, athletes and related staff needed to have heightened hygiene awareness. The *Epidemic Prevention Manual* included the principle of “hygiene awareness,” which mentioned keeping good hygiene awareness in mind- washing hands frequently, regularly disinfecting, avoiding touching one’s face, correctly wearing masks, disinfecting public goods before use, and regularly ventilating the environment to keep the air unobstructed.

This study provides a theoretical and quantitative basis for the formulation of the *Epidemic Prevention Manual for the Beijing 2022 Winter Olympics and Winter Paralympics*. It also provides a scientific reference for the formulation of prevention and control measures for other major international events during the COVID-19 pandemic. Quantifying the effectiveness of common prevention and control measures can highlight areas that could be adjusted in subsequent strategies. For example, with the increase in vaccine coverage and the emergence of variant strains with different characteristics, simulation can be conducted by modifying the values of relevant parameters in the model, providing a basis for optimizing the prevention and control measures for different scenarios. Another example is that if the policy of centralized management of entry personnel cannot be achieved, the model could be adjusted to identify which measures could be optimized to identify the source of infection in time, reduce the infection of local residents, and reduce or delay the epidemic peak so that the epidemic can be quickly and effectively controlled at a low cost, and the impact on social and economic development as well as people’s lives can be minimized. For example, measures such as increasing the frequency of nucleic acid testing and health monitoring, speeding up tracking and isolation, reducing contact, and accurately controlling cases compensate for the effect of centralized isolation of entry personnel.

The 2022 Beijing Winter Olympics and Winter Paralympics were successful. According to the official data of the events, from January 23, 2022, to the closing of the Winter Paralympics on March 13, 2022, a total of 16,092 Olympic-related personnel entered the country through the airport, with a total of 284 people testing positive at the airport. Moreover, 2,546,100 nucleic acid tests were conducted in the closed-loop, with 179 positive people testing positive for a total of 463 cases [27]. However, there was no cluster epidemic spread in the closed-loop. This showed that the closed-loop management was very effective and showed the effectiveness of relevant prevention and control measures formulated based on the results of this study.

However, this study had several limitations: (1) the main purpose of this study was to evaluate the effects of reducing contact and strict entry measures on the prevention and control of the spread of COVID-19. We did not review the attenuation of vaccine effectiveness over time and the differences in the effectiveness of different vaccines in our model; (2) this study does not subdivide people by category and nationality or consider the space–time process, including venues, accommodation, and transportation. These issues will be investigated in subsequent research. (3) As this study serves the formulation of the *Epidemic Prevention Manual* in its preparatory stage, it is impossible to accurately predict the actual situation of the events at the beginning of the study, so there are differences between the hypothetical scenario and the actual situations of the events, e.g., there are certain differences between the total number in the simulation and the actual number of Olympic-related personnel. The model assumes that the Olympic-related personnel enter the country at the same time, so the source of infection in the closed-loop is only introduced by the current entry, and it is assumed that there are fewer sources of infection introduced. However, in reality, after the opening of the Olympic Village, overseas Olympic-related personnel entered the country in batches, and each batch of entry personnel might bring the virus into the closed loop. The fact that there was no cluster epidemic spread during the events still verified the rationality of the conclusions of this study. We will continue to optimize the model further using the actual situation of the Winter Olympics and highlight ways that prevention and control measures could be optimized for hosting large-scale sports events in the future.

## Conclusions

Our results showed that daily nucleic acid testing, reducing effective contact between people, and health monitoring were the most important means to control the spread of the virus within a closed loop. The implementation of the measures also needs to be combined with the difficulty of implementation and the acceptance of the relevant personnel. The effective combination of various measures is the key to reducing the spread of the virus and ensuring the safe and wonderful holding of the Winter Olympics. Our research provided an important reference for formulating the key epidemic prevention and control measures for Beijing 2022 Winter Olympics and Winter Paralympics. The Beijing Winter Olympics and Winter Paralympics were a complete success, with no epidemic spread in the closed-loop during the games, which indirectly proves the rationality of our research results. This study details a number of ideas and methods that could be used for the formulation of prevention and control measures for other major international competitions and the optimization of subsequent strategies during the pandemic.

## Supplementary Information

**Additional file 1. **Scenario setting and evaluation index results.

## Data Availability

All data generated or analyzed during this study are included in this published article.

## Abbreviations

SARS-CoV-2: Severe acute respiratory syndrome coronavirus 2
SEIR: Susceptible-exposed-infected-recovered
Epidemic Prevention Manual: Epidemic Prevention Manual for the Beijing 2022 Winter Olympics and Winter Paralympics
