# Supplementary material for: Optimization of COVID-19 prevention and control measures during the Beijing 2022 Winter Olympics: a model-based study
Source: Infect Dis Poverty. 2022 Sep 6;11:95. doi: 10.1186/s40249-022-01019-2 (PMC9447360; doi:10.1186/s40249-022-01019-2)
Supplement: Supplementary file 1 — Additional file 1. Scenario setting and evaluation index results. [file 40249_2022_1019_MOESM1_ESM.docx]

# Methods

On non-testing day, the model can be expressed by the following differential equations:

When $t\neq t_{n}\left( t_{n}=t_{0}+nT \right)$($t_{0}$ is the initial time, $T$ is the interval between two nucleic acid tests, $t_{n}$ is the test time):

$$\begin{aligned} \frac{dS}{dt}=-\left( cp_{SC}\frac{I+A}{N}+\beta_{SE}\frac{P}{N} \right)S+\tau_{S}S_{q}\#\left( \text{1} \right) \end{aligned}$$

$$\begin{aligned} \frac{dS_{q}}{dt}=-\tau_{S}S_{q}\#\left( \text{2} \right) \end{aligned}$$

$$\begin{aligned} \frac{dV}{dt}=-\left( cp_{SC}\frac{I+A}{N}+\beta_{SE}\frac{P}{N} \right)V\left( 1-f_{S} \right)p+\tau_{V}V_{q}\#\left( \text{3} \right) \end{aligned}$$

$$\begin{aligned} \frac{dV_{q}}{dt}=-\tau_{V}V_{q}\#\left( \text{4} \right) \end{aligned}$$

$$\begin{aligned} \frac{dE}{dt}=\left( cp_{SC}\frac{I+A}{N}+\beta_{SE}\frac{P}{N} \right)S+\left( cp_{SC}\frac{I+A}{N}+\beta_{SE}\frac{P}{N} \right)V\left( 1-f_{S} \right)p-\theta\sigma_{I}E-\left( 1-\theta\right)\sigma_{A}E\#\left( \text{5} \right) \end{aligned}$$

$$\begin{aligned} \frac{dE_{q}}{dt}=-\delta E_{q}\#\left( \text{6} \right) \end{aligned}$$

$$\begin{aligned} \frac{dI}{dt}=\theta\sigma_{I}E-{\left( 1 - p_{I2R} \right)\delta}_{I}I-p_{I2R}\gamma_{I}I\#\left( \text{7} \right) \end{aligned}$$

$$\begin{aligned} \frac{dA}{dt}=\left( 1-\theta\right)\sigma_{A}E- p_{A2R}\gamma_{A}A\#\left( \text{8} \right) \end{aligned}$$

$$\begin{aligned} \frac{dH}{dt}=\left( 1 - p_{I2R} \right)\delta_{I}I+\delta E_{q}-\gamma_{H}H\#\left( \text{9} \right) \end{aligned}$$

$$\begin{aligned} \frac{dR}{dt}=\gamma_{H}H+p_{I2R}\gamma_{I}I+p_{A2R}\gamma_{A}A\#\left( \text{10} \right) \end{aligned}$$

$$\begin{aligned} \frac{dP}{dt}=\eta_{I}I+\eta_{A}A-\mu_{P}P\#\left( \text{11} \right) \end{aligned}$$

Among them, $t^{-}$ represents the time before nucleic acid testing, and $t^{+}$ represents the time after testing, tracking and isolation of close contacts.

## Results

The sensitivity analysis in the main text showed that the most important measures (in descending order) were daily nucleic acid testing, reducing contact among people, and daily health monitoring. Figure S1 demonstrates the effect of random combinations of individual effective measures on controlling the COVID-19 outbreak in the Winter Olympics. The results show that each combination of measures has a significant effect on preventing the spread of the virus. Specifically, the combination of the three measures had the best effect on epidemic control, with fewer than 5 people infected during the simulation period, followed by combinations “daily nucleic acid testing + reducing contact among people”, “daily nucleic acid testing + daily health monitoring”, and “reducing contact among people + daily health monitoring”, which also verifies the conclusions of the sensitivity analysis.


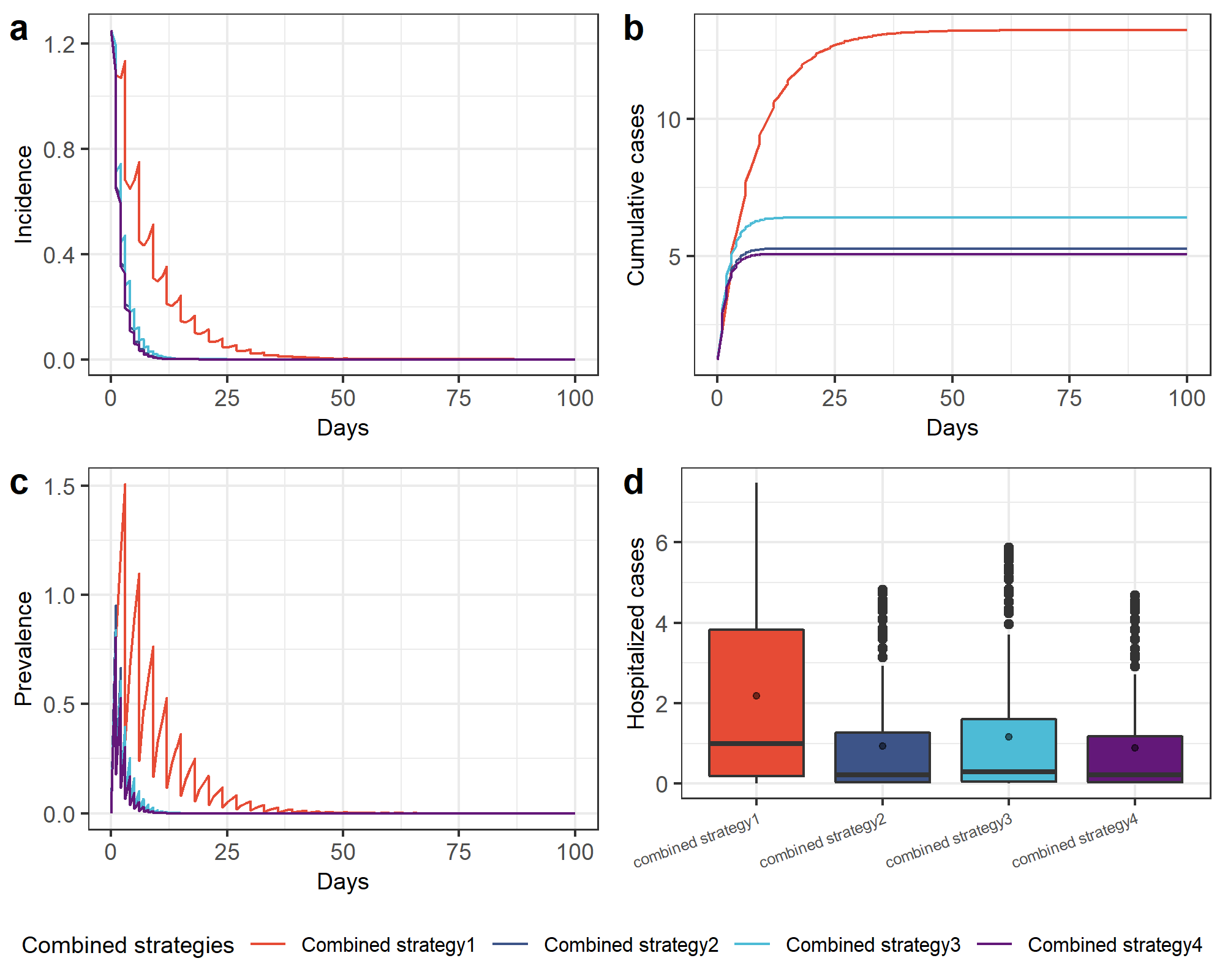


Figure S1. **Simulation results of COVID-19 transmission in combined interventions strategies.** a, incidence over time; b, cumulative cases over time; c, prevalence over time; d, hospitalized cases. Boxplots represent the mean (dots), median (black line), quarter and third quartile (upper and lower edges of the box), maximum (upper edge) and minimum (lower edge) of hospitalized cases for each scenario. Combined strategy1 represents “reducing contact among people + daily health monitoring”, combined strategy2 represents “daily nucleic acid testing + reducing contact among people”, combined strategy3 represents “daily nucleic acid testing + daily health monitoring”, and combined strategy4 represents “daily nucleic acid testing + reducing contact among people + daily health monitoring”. In Figure S1 a, the curves of combined strategy2 and combined strategy4 basically coincide.

TableS1 Scenario setting and evaluation index results

| Scenarios | | Parameter value | Description | Peak of incidence | Peak of prevalence | Peak time | Cumulative cases | Peak of hospitalized cases |
| --- | --- | --- | --- | --- | --- | --- | --- | --- |
| S1 | Baseline | $E_{0}=5,A_{0}=0, I_{0}=0$;  $cp_{SC}=5$, $\beta_{SE}=0.00414$,  $n=50;$  $\eta_{A}=0.05$, $\eta_{I}=0.1$, $\mu_{P}=0.2;$  $\delta_{I}=1/2,k=3$ | See description below | 477.53 | 750.62 | 57 | 13382.73 | 6746.03 |
| S2 | Relax entry measures | $E_{0}=10,A_{0}=5, I_{0}=0$ | $E_{0}$: number of initial exposed; $A_{0}$: number of initial asymptomatic infected; $I_{0}$: number of initial symptomatic infected | 484.02 (↑1.34%) | 761.81(↑1.49%) | 42 | 13566.65(↑1.4%) | 6825.46(↑1.18%) |
| S3 | Strict entry measures | $E_{0}=2,A_{0}=0, I_{0}=0$ |  | 475.31(↓0.46%) | 750.5(↓0.02%) | 66 | 13180.21 (↓1.51%) | 6730.02 (↓0.24%) |
| S4 | Increase contact | $cp_{SC}=7.5$,$\beta_{SE}=0.00621$, $n=75$ | $cp_{SC}$: the effective contact rate between people;  $\beta_{SE}$: the effective contact rate between people and the environment;  $n$: number of close contacts per infected person | 1517.84(↑214.93%) | 2354.41 (↑213.66%) | 30 | 22264.37(↑66.4%) | 16070.87(↑138.23%) |
| S5 | Reduce contact | ${cp}_{SC}=2.5$,$\beta_{SE}=0.00207$, $n=25$ |  | 1.30 (↓99.72%) | 2.16(↓99.71%) | 3 | 19.21 (↓99.86%) | 10.45 (↓99.85%) |
| S6 | High environmental risk | $\eta_{A}=0.075$, $\eta_{I}=0.15$, $\mu_{P}=0.1$ | $\eta_{A}$: the rate at which asymptomatic infected individuals shed virus into the environment; $\eta_{I}$: the rate at which symptomatic infected individuals shed virus into the environment; $\mu_{P}$: Virus mortality in the environment | 478.28(↑0.15%) | 751.67 (↑0.14%) | 57 | 13400.36(↑0.13%) | 6829.75 (↑1.24%) |
| S7 | Low environmental risk | $\eta_{A}=0.025$, $\eta_{I}=0.05$, $\mu_{P}=0.3$ |  | 477.18(↓0.07%) | 750.14(↓0.06%) | 57 | 13376.29 (↓0.05%) | 6752.92(↓0.10%) |
| S8 | Cases not detected in time | $\delta_{I}=1/3, k=5$ | $1/\delta_{I}$: the time interval from having infectious ability to detecting infection through health monitoring for a symptomatic infected person; $k$: frequency of nucleic acid testing | 2053.47(↑330.02%) | 4953.73 (↑559.95%) | 30 | 29968.54(↑123.9%) | 18772(↑178.27%) |
| S9 | Cases detected in time | $\delta_{I}=1, k=1$ |  | 1.25(↓99.74%) | 0.84(↓99.88%) | 1 | 5.42(↓99.96%) | 5.87(↓99.91%) |
